# Qualitative and quantitative validation of the FACIT-fatigue scale in iron deficiency anemia

**DOI:** 10.1186/s12955-015-0257-x

**Published:** 2015-05-17

**Authors:** Sarah Acaster, Rene Dickerhoof, Kendra DeBusk, Kristine Bernard, William Strauss, Lee F. Allen

**Affiliations:** Oxford Outcomes, 188 Embarcadero, Suite 200, San Francisco, CA 94105 USA; AMAG Pharmaceuticals, 1100 Winter St., Waltham, MA 02451 USA

**Keywords:** Iron deficiency anemia, FACIT-fatigue, Fatigue, Psychometric validation, Content validity

## Abstract

**Background:**

Fatigue is a burdensome symptom in iron deficiency anemia (IDA). To capture the severity and impact of fatigue appropriately it must be measured using validated scales. This study evaluated the content validity and psychometric validity of the Functional Assessment of Chronic Illness Therapy - fatigue scale (FACIT-fatigue) in IDA patients.

**Methods:**

Qualitative patient interviews were conducted in the United States to evaluate content validity. The psychometric properties of the FACIT-fatigue scale were investigated using data from a phase 3 clinical trial assessing ferumoxytol in patients with a history of unsatisfactory oral iron therapy or in whom oral iron cannot be used. The statistical analysis assessed the acceptability, reliability, validity and responsiveness of the FACIT-fatigue scale.

**Results:**

Qualitative interviews showed that fatigue is a central concern to IDA patients and that the FACIT-fatigue scale sufficiently assessed this construct. Psychometric assessment demonstrated that the FACIT-fatigue scale was stable over time (ICC = 0.87) and internally consistent (α = 0.93). The scale demonstrated convergence with other conceptually relevant scales such as SF-36 Vitality (r = 0.74), and distinguished between known groups [i.e., treatment arms (mean difference (95 % CI) = 3.56 (1.68, 5.43), p <0.001) and high vs. low hemoglobin groups (mean difference (95 % CI) = 5.51 (8.59, 2.44) p <0.001)]. Responsiveness was also demonstrated; significant improvements in FACIT-fatigue scale scores corresponded with significant differences between minimal, moderate, and much improved vitality cohorts (*p* < 0.05).

**Conclusions:**

This research demonstrated that the FACIT-fatigue scale has sound measurement properties and is an appropriate and interpretable assessment of fatigue among IDA patients with various underlying conditions.

## Background

The World Health Organization (WHO) and the U.S. Center for Disease Control and Prevention define anemia as a hemoglobin value <12.0 g/dL in women and <13.0 to 13.7 g/dL in men [1, 2]. According to WHO estimates two billion people worldwide, or 30 % of the world’s population, are anemic [3, 4]. Iron deficiency anemia (IDA), caused by various underlying conditions such as abnormal uterine bleeding (AUB), post partum bleeding, cancer and gastro-intestinal (GI) disorders, is the leading cause of anemia worldwide; estimates suggest that approximately five million people have IDA in the United States [5].

Common symptoms of IDA include fatigue and exercise-associated dyspnea, poor mental performance and cold intolerance [6, 7]. Research involving anemic cancer patients has found an increase in hemoglobin is associated with an improvement in fatigue, which in turn is associated with improvements in health related quality of life (HRQL) [8, 9]. There is also evidence that decreases in hemoglobin are related to increases in fatigue duration [10].

The Functional Assessment of Chronic Illness Therapy - fatigue (FACIT-fatigue) scale [11, 12] is a 13-item instrument designed to assess fatigue/ tiredness and its impact on daily activities and functioning in a number of chronic diseases. The instrument includes items such as tiredness, weakness, listlessness, lack of energy, and the impact of these feelings on daily functioning (e.g., sleeping, and social activities). The FACIT-fatigue scale (previously called the Functional Assessment of Cancer Therapy-Fatigue (FACT-F)) was originally developed to assess cancer-related fatigue and has shown good reliability and validity in a sample of cancer patients [12]. More recently the content validity and / or psychometric properties of the instrument have been established in numerous chronic conditions such as systemic lupus erythematosus [13, 14]; rheumatoid arthritis [15]; psoriatic arthritis [16]; chronic immune thrombocytopenia [17] and Parkinson’s disease [18]. However, to date, the content validity and psychometric properties have not been tested or demonstrated in IDA. In order to use the instrument to evaluate the impact of new treatments for IDA on levels of fatigue or the impact of fatigue it is necessary to demonstrate that the instrument is a reliable and valid measure of fatigue for patients with IDA.

Many validation studies focus on the psychometric properties only, without also exploring the content validity of an instrument. Content validity is the extent to which an instrument contains the relevant and important aspects of the concept of interest, and is specific to the population of interest [19, 20]. Evidence of content validity is required if a patient-reported outcome (PRO) instrument is used to support a labeling claim for a new product in the United States [19]. Evidence is obtained by analyzing how the content of the instrument relates to the construct it is intended to measure. In order to do this, qualitative research with the population of interest can be conducted to explore whether the PRO instrument covers the concept of interest [20]. Therefore this paper reports research aimed to assess both the content validity and psychometric properties of the FACIT-fatigue scale in patients with IDA. Throughout the paper the qualitative content validity methods and results are presented before the psychometric, quantitative, methods and results. This reflects the fact that psychometric evidence is necessary but not sufficient to support validity; qualitative content validity is also required.

## Methods

The qualitative content validity study was conducted as a stand alone cross sectional study in 2013. The data utilized for the psychometric validation analysis was from a clinical trial that was completed before the qualitative study was conducted (2012). As such the study design and participants section below details each study in turn. The inclusion / exclusion criteria for the qualitative study were designed to reflect those used in the clinical trial to minimize any potential differences between the study populations.

### Study design and participants

#### Qualitative content validation

A cross sectional, qualitative study design was developed to gather information on the nature of fatigue and its impact on HRQL experienced by IDA patients with various underlying conditions. A two-stage concept elicitation-cognitive debrief semi-structured interview format was developed. To reduce bias introduced when prompting for concepts/symptoms associated with IDA, the concept elicitation portion of the interview was conducted first. Patients were asked to describe their anemia symptoms and the impact of these symptoms on their functioning. If fatigue was not spontaneously reported during the concept elicitation interview, the interviewer asked the patient if they experienced fatigue before moving on to the cognitive debrief interviews. During the cognitive debrief interview patients completed a copy of the FACIT-fatigue scale and were asked to “think aloud”-that is, to vocalize their interpretations of and any problems with the instructions, items, recall period, and response options. This approach permitted an evaluation of the relevance, interpretability, clarity and ease of understanding of all concepts and content of the FACIT - fatigue scale. To keep the patient engaged throughout this process, the interviewer was prepared with numerous probes that fostered a conversation-like environment. Each two-stage interview lasted approximately 60 min.

Participant recruitment was conducted through three clinical sites in the United States. A purposive sampling strategy was used in order to include patients with different underlying conditions including, AUB, post partum bleeding and GI disorders. While cancer is also a potential underlying condition of IDA, a cancer specific population was involved in the original development of the FACIT-fatigue and therefore not prioritized [12]. Patients were eligible to take part if: they were at least 18 years of age; they had a clinical diagnosis of IDA [measurable hemoglobin (Hgb) range of > 7 g/dL to < 10 g /dL and transferrin saturation (TSAT) < 20 %]; they were able to speak and read English and provide written informed consent. Patients were ineligible if they fulfilled any of the Phase 3 trial exclusion criteria detailed in the Psychometric Validation Section below.

#### Psychometric validation

The psychometric validation was conducted on the intent-to-treat population from a Phase 3 randomized, placebo-controlled, double-blind trial, which examined the impact of intravenous (IV) ferumoxytol on HRQL compared to IV placebo in IDA patients who had a history of unsatisfactory oral iron therapy or in whom oral iron could not be used (ClinicalTrials.gov identifier: NCT01114139).

The trial consisted of a Screening Period of up to 2 weeks followed by a 5-week Treatment Period. The 5-week Treatment Period consisted of six study visits: Day 1 (Baseline, Dose 1), Week 1 (Dose 2; 2 to 8 days post-Dose 1), and weekly thereafter up to Week 5. Patients received a Baseline (Day 1) IV injection of either ferumoxytol 510 mg (17 mL) or normal saline administered as a rapid IV injection in under 1 min with a second dose 2 to 8 days later. The FACIT-Fatigue questionnaire was administered at Baseline and every week thereafter to Week 5. The SF-36 and LASA questionnaires were administered at Baseline, Week 3, and Week 5 only.

Eligible patients were males and females ≥18 years of age with a history of IDA defined as a Hgb <10.0 g/dL and a TSAT <20 %, who also had a history of unsatisfactory oral iron therapy or in whom oral iron therapy could not be used. Patients were not eligible for participation if they had a history of allergy to IV iron, a Hgb ≤7.0 g/dL, serum ferritin >600 ng/mL, known causes of anemia other than iron deficiency, active infection, hematologic malignancies, were on dialysis or had an estimated glomerular filtration rate <30 mL/min/1.73 m^2^, or were pregnant, intended to become pregnant, or were breastfeeding. Patients who received another investigational agent or parenteral iron therapy within 4 weeks of screening or who had received oral iron therapy or blood transfusion within 2 weeks prior to screening were also excluded. A full description of the clinical trial is reported elsewhere [21].

### Ethics

Full Institutional Review Board (IRB) approval was obtained from the Independent IRB Inc. (IIRB, Plantation, Florida) prior to beginning recruitment of participants for the qualitative study. The clinical trial used for the psychometric validation analysis was conducted according to Good Clinical Practice (GCP) guidelines and in compliance with the ethical principles of the Declaration of Helsinki, and was approved by the ethics committee or institutional review board of each participating center prior to the commencement of the study. All patients in both studies provided written informed consent.

### Measures

Three PRO measures were used: the FACIT-fatigue [12], the Medical Outcomes Survey Short Form – 36 (SF-36) [22], and the Linear Analogue Scale Assessments (LASA) [23]. The FACIT-fatigue was used in both the qualitative interviews and psychometric validation; the SF-36 and LASA were used in the psychometric validation only.

The FACIT-fatigue scale is a 13-item patient-reported measure of fatigue with a 7-day recall period. Items are scored on a 0 – 4 response scale with anchors ranging from “Not at all” to “Very much so”. To score the FACIT-fatigue, all items are summed to create a single fatigue score with a range from 0 to 52. Items are reverse scored when appropriate to provide a scale in which higher scores represent better functioning or less fatigue.

The SF-36 is a validated generic HRQL instrument intended for use in a wide range of conditions as well as the general population that can be self-administered. 36 items assess patient health across eight domains: bodily pain (BP), general health perceptions (GH), mental health (MH), physical functioning (PF), role limitations due to emotional functioning (RE), role limitations due to physical functioning (RP), social functioning (SF), and vitality (VT). All items use categorical response options (range: 2 – 6 options). From the individual subscales, two component summary scores are generated for physical (PCS) and mental health (MCS). The first five subscales (PF, RP, BP, GH, VT) produce the PCS and the last five subscales (GH, VT, SF, RE, MH) produce the MCS; the GH and VT subscales overlap between the two overall components. The scores for each subscale are converted to norm-based scores (based on 1998 US general population), with a mean of 50 and a standard deviation of 10. A score of 100 represents the best health. The validity and reliability of the SF-36 has been well established [24].

The LASA consists of three visual analogue scales (VAS), one for each of the following domains: Energy Level, Activities of Daily Living (ADL), and Overall Quality of Life (QOL). Each VAS has a 7-day recall period and consists of a 100-mm line with a left anchor representing the worst possible score (0) and the right anchor representing the best possible score (100). Higher scores are indicative of better functioning / HRQL. VAS scales have been established as valid and reliable PRO tools [25].

### Data analysis

#### Qualitative content validation

All interviews were audio recorded and transcribed verbatim for qualitative analysis. Thematic analysis [26, 27] was used for the concept elicitation interviews and content analysis for the cognitive debrief analysis [28]. The qualitative software tool MAX QDA was used to assist the analysis. Inductive coding was used to identify themes in the concept elicitation data. In order to minimize potential bias approximately 15 % of the transcripts were ‘double coded’ by a second qualitative researcher. The ‘double coded’ transcripts were reviewed by a third researcher and any discrepancies were resolved in a meeting between all three researchers. Saturation, which can be defined as data adequacy or the point at which no new information is obtained from additional qualitative data [29] was assessed using saturation tables [30].

#### Psychometric validation

The measurement properties of the FACIT-fatigue were evaluated using four standard statistical assessments: data acceptability, reliability, validity, and responsiveness.

- Data acceptability was determined by examining score distributions; acceptability is supported when observed scores are well distributed, and mean scores are near the scale mid-point [31]. At the item level, some items should demonstrate a skewed distribution of response to reflect the full range of severity of the concept is covered. Data acceptability was assessed at Baseline and Week 3 in case the trial inclusion criteria created a restriction in range of scores.
- Reliability was assessed by evaluating internal consistency and test re-test reliability. Internal consistency reliability was determined using Cronbach’s alpha (α) coefficient [32] and item to total correlations: an α ≥ 0.80 [33, 34] and item to total correlations ≥ 0.20 [35] were used as a guide for determining that the FACIT-fatigue was internally consistent. Test re-test reliability was evaluated using an intra-class correlation coefficient (ICC). As test re-test reliability is designed to evaluate stability over time, this analysis was conducted on a subset of patients with stable Hgb (<0.5 g/dL change) from Week 3 to Week 4. An ICC of ≥ 0.80 was used as a guide to determine test re-test reliability [36].
- Validity assesses the extent to which a scale measures or correlates with the concept it purports to measure. Validity was determined based on correlations between the FACIT-fatigue and other related PRO scales (SF-36 and LASA), and known groups comparisons. While fatigue is likely to be related to all aspects of HRQL it was hypothesized that the SF-36 VT and RP domains and LASA Energy and ADL domains would demonstrate the highest correlations at Baseline. Known groups comparisons were explored based on Hgb levels (high vs. low) and treatment arm comparisons (ferumoxytol vs. placebo). It was hypothesized that patients with high Hgb (Hgb > 12 g/dL) and patients in the active treatment arm would have higher FACIT-fatigue scores (less fatigue) than patients with low Hgb (Hgb < 9 g/dL) or in the placebo arm at Week 3.
- Responsiveness was determined based on change from Baseline to Week 3 in Hgb level and SF-36 VT score. Three Hgb groups were created: improved (≥1 g/dL), stable (0 - <1 g/dL) and worsened (<0 g/dL). Five SF-36 VT groups were created: much improved (≥20), moderately improved (10 - <20), minimally improved (5 - <10), stable (0 –<5) and worsened (<0). Change from Baseline FACIT-fatigue score within each group was assessed using repeated samples t-tests; differences between change groups were assessed using Tukey’s post-hoc pairwise comparisons.

All analyses were conducted on the psychometric validation sample as a whole, unless otherwise specified, using SPSS version 20.0.

## Results

### Patient Characteristics

Patient characteristics for the qualitative content validity and psychometric validity samples are presented in Table 1. The psychometric validation sample included 808 patients (intent-to-treat (ITT) population) who had any exposure to study drug (ferumoxytol, n = 608; placebo, n = 200) and excluded 4 patients who withdrew from the study prior to administration of study drug. Baseline demographic and clinical characteristics, and FACIT-fatigue scores were comparable across treatment groups.

**Table 1:** Patient demographics and clinical history

Baseline characteristics	Psychometric Validation Sample		Qualitative Content Validation Sample
	Ferumoxytol (n = 608)	Placebo (n = 200)	
Age, years, mean (± SD)	44.8 (±13.82)	46.0 (±13.58)	41.7 (±11.3)
Female, n (%)	542 (89.1)	178 (89.0)	14 (93.3)
Race, n (%)			
Asian	98 (16.1)	32 (16.0)	-
Black/African American	152 (25.0)	50 (25.0)	11 (73.3)
White	340 (55.9)	111 (55.5)	4 (26.7)
Other/Multiracial	18 (3.0)	7 (3.5)	-
Hgb g/dL, mean (± SD)	8.9 (±0.89)	8.8 (±0.89)	-
TSAT, mean (± SD)	7.0 (12.9)	5.4 (4.9)	-
Underlying condition, n (%)			
AUB	260 (42.8)	84 (42.0)	11 (73.3)
Cancer	29 (4.8)	10 (5.0)	-
GI disorders	173 (28.5)	58 (29.0)	1 (6.7)
Postpartum anemia	4 (0.7)	0 (0.0)	2 (13.3)
Other ^a^	142 (23.4)	48 (24.0)	1 (6.7)
FACIT-fatigue, mean (± SD)	24.1 (11.8)	24.7 (11.3)	-

SD = Standard Deviation; n = sample size; Hgb = hemoglobin; TSAT = transferrin saturation; AUB = abnormal uterine bleeding; GI = gastro-intestinal

^a^ Other included nutritional iron deficiency, heart failure, and rheumatoid arthritis

The content validation sample included 15 patients; age and sex characteristics were comparable to the psychometric sample and almost all key IDA sub groups were included, with the exception of cancer. Due to the cognitive debrief component of the qualitative analysis patients level of education was also recorded for this sample; this ranged from less than high school education to graduate degree.

### Content validity

#### Concept elicitation

All patients spontaneously reported that their main symptom experiences were “fatigue” (N = 8), “tiredness” (N = 13), and / or “low energy” (N = 8). Other symptoms/concepts reported included feeling weak (N = 2), being cold (N = 2), excessive sweating (N = 2), dark circles around the eyes (N = 2), restless (N = 1), difficulty sleeping (N = 1), dizziness (N = 1), light-headedness (N = 1), chest pains (N = 1), nausea (N = 1), muscle aches (N = 1), and hair loss (N = 1). All Patients also reported that their symptoms had a significant impact on daily functioning (i.e., on their home and social activities). Six patients specifically reported that they needed to sleep during the day, and that they viewed this problem as a separate concept from the impact of fatigue on their daily activities. Evaluation of data saturation established that no new themes were added after the 4th interview. All underlying condition subgroups spontaneously reported fatigue / tiredness / low energy as a primary symptom that impacted their HRQL and therefore further concept saturation within each sub group was not deemed necessary given the aim of this study.

#### Cognitive debrief

The cognitive debrief findings are summarized in Table 2. In general, patients were able to understand and interpret the FACIT-fatigue scale instructions, items, response options, and recall period without any problems. Further, most patients (n = 12) felt that every item on the FACIT-fatigue scale was relevant to their experience of IDA; item #10 (“Too tired to eat”) received the least endorsement yet was still relevant to the majority (12/15; 80 %). When asked whether other important concepts related to their experience of IDA were not represented on the FACIT-fatigue scale, all patients reported that the most important concepts were present.

**Table 2:** Content validity: cognitive debrief summary

FACIT-fatigue Item	Correctly Interpreted	Relevant to IDA	Example Quotes
1. I feel fatigue	14/15	14/15	“I feel tired and worn out… your body’s like - ugh…”
2. I feel weak all over	15/15	14/15	“I get really weak and drained… like someone just sucked all the energy out of you” “I just take my time moving around, and work at getting myself built back up”
3. I feel listless (washed out)	14/15	13/15	“Feeling lifeless and you don’t want to do anything”
4. I feel tired	15/15	15/15	“I just want to sit back on the couch and do nothing -just you know lack of energy”
5. I have trouble starting things because I am tired	15/15	15/15	“So I had to get up and force myself…So literally you have to like mentally coach, coax myself to move forward.”
6. I have trouble finishing things because I am tired	15/15	15/15	“I will go lay down…I say I will do it later, and I might not get back to it”
7. I have energy	15/15	15/15	“I have energy would mean, you know, I don’t feel tired, you know … I get up and I have no problem getting started, I go through the day doing whatever I have to do and I can go, keep going, you know… I’m not describing myself”
8. I am able to do my usual activities	15/15	15/15	“No, I’m not able to do my usual stuff”… “I need help with the yard work…”
9. I need to sleep during the day	15/15	15/15	“I’ll sleep during the day—if I have to run errands, if I’m taking my husband to an appointment, I literally fall asleep, you know, sitting in the chair waiting—anywhere I can take a nap, I’ll take a nap.”
10. I am too tired to eat	15/15	12/15	“I get that way sometimes. Typically if I get that way it’s because…I’m running out of energy by that time”.
11. I need help doing my usual activities	15/15	13/15	“…sometimes I do get help from my husband and then even times at work, like close colleagues, I will receive assistance from them”
12. I am frustrated by being too tired to do the things I want to do	15/15	15/15	“Yeah, right, because you know, who would think to actually get like frustrated because you’re tired. Yeah, I am. I don’t think people realize that it’s something that you can’t really control.”
13. I have to limit my social activity because I am tired	15/15	15/15	“with the last date I went, I think, my attention span began to wane because I was tired. I was like— oh, my God, you’re sitting there thinking I want to go home.”

### Psychometric validity

#### Data acceptability

As shown in Table 3 data acceptability was supported. At both time points all response options were endorsed, and the mean and median item values were reasonably comparable. Some items demonstrated evidence of floor and ceiling affects, demonstrating the full range of fatigue severity is captured.

**Table 3:** FACIT-fatigue scale acceptability and internal consistency reliability data at Baseline and Week 3

FACIT-fatigue Item	Baseline (n = 792)					Week 3 (n = 738)				
	Score Range (0 –4)	Mean / Median (SD)	Floor /Ceiling %	α	Item - total Correlation	Score Range (0 –4)	Mean / Median (SD)	Floor /Ceiling %	α	Item - total Correlation
I feel fatigue	0 – 4	2.71 / 3.0 (1.15)	4.3 / 29.1	0.92	0.73	0 – 4	1.61 / 1.0 (1.12)	14.9 / 5.2	0.94	0.81
I feel weak all over	0 – 4	2.26 / 2.0 (1.25)	11.4 / 17.3	0.92	0.77	0 – 4	1.23 / 1.0 (1.14)	31.1 / 3.7	0.94	0.82
I feel listless (washed out)	0 – 4	2.31 / 2.0 (1.29)	12.4 / 19.9	0.92	0.79	0 – 4	1.21 / 1.0 (1.18)	33.3 / 4.2	0.94	0.86
I feel tired	0 – 4	2.85 / 3.0 (1.12)	3.3 / 33.7	0.92	0.75	0 – 4	1.71 / 2.0 (1.15)	12.4 / 7.8	0.94	0.81
I have trouble starting things	0 – 4	2.31 / 2.0 (1.27)	11.5 / 20.2	0.92	0.81	0 – 4	1.28 / 1.0 (1.15)	29.0 / 3.3	0.94	0.85
I have trouble finishing things	0 – 4	2.30 / 2.0 (1.28)	12.3 / 19.7	0.92	0.82	0 – 4	1.34 / 1.0 (1.19)	28.5 / 3.7	0.94	0.84
I have energy	0 – 4	1.41 / 1.0 (0.98)	17.2 / 2.7	0.93	0.48	0 – 4	1.96 / 2.0 (1.03)	6.2 / 5.9	0.94	0.53
I am able to do my usual activities	0 – 4	2.14 / 2.0 (1.02)	5.0 / 9.4	0.93	0.51	0 – 4	2.51 / 3.0 (1.02)	2.2 / 16.3	0.95	0.50
I need to sleep during the day	0 – 4	2.10 / 2.0	13.9 / 17.9	0.93	0.57	0 – 4	1.32 / 1.0 (1.19)	27.4 / 5.2	0.94	0.61
I am too tired to eat	0 – 4	1.06 / 1.0 (1.31)	42.2 / 2.7	0.93	0.51	0 – 4	0.57 / 0.0 (0.91)	59.7 / 0.7	0.94	0.56
I need help doing my usual activities	0 – 4	1.14 / 1.0 (1.14)	39.2 / 3.7	0.93	0.58	0 – 4	0.79 / 0.0 (1.02)	48.9 / 1.6	0.94	0.63
I am frustrated	0 – 4	2.29 / 2.0 (1.17)	16.2 / 29.1	0.92	0.77	0 – 4	1.32 / 1.0 (1.32)	33.4 / 7.9	0.94	0.81
I have to limit my social activity because I am tired	0 – 4	2.02 / 2.0 (1.45)	16.7 / 17.0	0.92	0.78	0 – 4	1.18 / 1.0 (1.13)	31.9 / 3.6	0.94	0.82

Score: 0 = ‘Not at all’, 1 = ‘A little bit’, 2 = ‘Somewhat’, 3 = ‘Quite a bit’, 4 = ‘Very much’; Floor = % responding ‘0’; Ceiling = % responding ‘4’; α = Cronbach’s alpha with the item removed

#### Reliability

Internal consistency reliability was good: Cronbach’s α = 0.93. As shown in Table 3 no single item significantly impacted the overall scale internal consistency and all items correlated well with the overall score. Test re-test reliability was also good with an ICC of 0.87 reported across Weeks 3 and 4 amongst Hgb stable patients.

#### Validity

As hypothesized the FACIT-fatigue scale was most highly correlated with the SF-36 VT domain (*r* = 0.74), and LASA Energy (*r* = 0.71) and ADL (*r* = 0.71) domains. The LASA QOL and SF-36 RP and SF domains all showed similar correlations with the FACIT-Fatigue (r = 0.68, 0.67, 0.66, respectively). The SF-36 MCS and PCS domains showed slightly lower levels of association with the FACIT-fatigue (*r* = 0.62 and 0.59, respectively); all other domains were correlated between *r* = 0.50 – *r* = 0.54 with BP and GH providing the lowest correlations.

The validity of the FACIT-fatigue was further supported by the known groups validity data. As shown in Table 4 patients with higher Hgb levels and patients receiving active treatment rather than placebo reported significantly lower levels of fatigue (higher FACIT-fatigue scores) at Week 3.

**Table 4:** Known groups analysis: comparison of hemoglobin level groups and treatment arms at Week 3

FACIT-fatigue WK 3	Hemoglobin (Hgb) Severity		Treatment Arm	
	High Hgb (>12 g/dL)	Low Hgb (< = 9 g/dL)	Ferumoxytol	Placebo
N	102	106	555	183
Mean	35.96	30.45	35.78	32.22
SD	10.82	11.64	11.31	10.92
Mean Difference (95 % CI)	-5.51 (-8.59, -2.44)		- 3.56 (-1.68, -5.43)	
P value	<0.001		<0.001	

#### Responsiveness

**Fig. 1:**
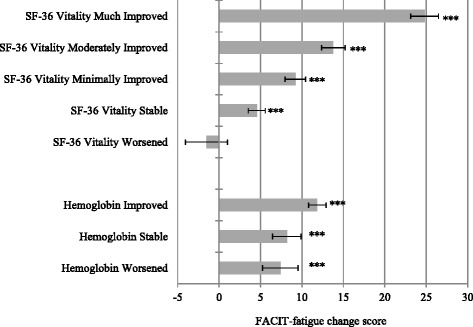
FACIT-fatigue responsiveness: change from Baseline to Week 3 by hemoglobin and SF-36 Vitality domain. Hemoglobin groups: improved (≥1 g/dL), stable (0 - <1 g/dL) and worsened (<0 g/dL). SF-36 VT groups: much improved (≥20), moderately improved (10 - <20), minimally improved (5 - <10), stable (0 – <5) and worsened (<0)*** Change from Baseline to Week 3 p < 0.001 Error bars represent 95 % confidence intervals (1.96 x standard error)

As illustrated in Fig. 1, the FACIT-fatigue demonstrated good responsiveness / ability to detect change. Changes in the FACIT-fatigue directly reflected changes in the SF-36 VT domain from Baseline to Week 3. All improved groups demonstrated significant change from Baseline, while the stable group also demonstrated significant improvement from Baseline the mean FACIT-fatigue change (4.56) was in line with the definition of SF-36 VT stability (0 – < 5). The worsened group did demonstrate a decline in FACIT-fatigue score, but this did not reach statistical significance. However, Tukey’s post-hoc paired comparisons found statistically significant (p < 0.05) differences between all SF-36 VT change groups.

The hemoglobin results demonstrated lower responsiveness as all groups showed significant improvement in FACIT-fatigue scores from Baseline to Week 3. However, Tukey’s post hoc paired comparisons did find significantly higher FACIT-fatigue improvement in the improved Hgb group compared to the stable and worsened groups (*p* < 0.05). There was no significant difference between the stable and worsened Hgb groups.

## Discussion

This study assessed the qualitative content validity and psychometric validity of the FACIT-fatigue scale in IDA patients with various underlying conditions. The content validity data supported fatigue as a primary symptom among this patient population; all patients spontaneously reported experiencing fatigue, tiredness and / or low energy. In addition, the cognitive debrief of the FACIT-fatigue found that the majority of patients (80 –100 %) correctly interpreted each item and felt each item to be relevant to their experiences. The item considered least relevant was feeling ‘too tired to eat’; this is in line with findings from other validation studies e.g. [13] and is likely to reflect the very severe nature of the item’s content. However, even this severe item was still identified as appropriate by the majority of patients (80 %). It should be noted that while the qualitative study sample was a reasonable reflection of the psychometric study sample, no IDA patients with cancer as their underlying condition were included in the qualitative sample. However, as the FACIT was originally developed for a cancer population, this was not considered a limitation of the study.

In line with the content validity evidence, the psychometric validity analyses further supported the appropriateness of the FACIT-fatigue within IDA populations. The data acceptability results demonstrated good variability across responses. All response options were utilized for all items, mean and median values were similar and there was evidence of skewed distribution for items at opposite ends of the severity spectrum. In line with the qualitative findings the ‘too tired to eat item’ did demonstrate greater floor effects (high proportion of ‘not at all’ responses) at Baseline and Week 3 than any other item. As it is important for a scale to capture the full spectrum of severity in order to permit responsiveness to decline and improvement, this should not be considered a weakness of the FACIT-fatigue in this population.

In addition to demonstrating good data acceptability, the descriptive item level findings also demonstrated the responsiveness of the FACIT-fatigue scale; the direction of severity was shown to improve from Baseline to Week 3 as would be expected in a trial involving active treatment. This was further supported in the specific responsiveness analysis, which demonstrated incremental improvement and decline in line with the SF-36 VT domain, and hemoglobin improvement to a lesser extent. Ideally, responsiveness would have been explored using a patient global impression of concept (fatigue) measure across time points (e.g. asking patients to rate their global impression of fatigue at Baseline and Week 3 and comparing the FACIT-fatigue scores according to their global change). However, as the psychometric validation study was conducted as *post-hoc* analysis of trial data, rather than being a stand-alone psychometric validation study, the use of the SF-36 VT and hemoglobin groups as suitable anchors of change was considered appropriate. The correlation between the FACIT-fatigue and SF-36 VT domain (*r* = 0.74) supported this assumption [37]; the lower levels of responsiveness associated with hemoglobin as a biomarker versus a comparable patient-reported outcome measure was not unexpected. One interpretation of these findings is that that hemoglobin levels alone do not fully explain the experience of fatigue in IDA populations and therefore demonstrate the importance of capturing the patient experience of fatigue as well as this biomarker in clinical trials of IDA. However, as patients entering this study had suppressed hemoglobin and therefore potentially suppressed fatigue, these changes may reflect regression to the mean rather than responsiveness. Further, in the case of the stable and worsened groups, improved fatigue may reflect placebo effects.

In relation to the reliability of the FACIT-fatigue, test-retest reliability analysis demonstrated ‘very good’ reproducibility, and the Cronbach’s alpha assessment showed ‘good’ internal consistency reliability. Furthermore, the item-total correlations all exceeded suggested guidelines, and the removal of any individual item had no impact on the internal consistency. This suggests that all FACIT-fatigue scale items relate to the global concept under assessment and that no items should be removed to improve the internal consistency of the scale.

The hypothesized convergent validity associations were largely supported; as predicted, the SF-36 VT and LASA Energy and ADL items were the most strongly correlated constructs to the FACIT-fatigue scale. However, while the SF-36 RP was also strongly correlated to the FACIT-fatigue (as predicted), this was also true of the SF-36 SF and LASA QOL domains. Thus, while correlational convergence between fatigue and conceptually related PRO constructs was seen, all aspects of HRQL were associated with fatigue to a moderate extent. Given the conceptual link between social and mental functioning with fatigue, these results do not challenge the evidence of good convergent validity. Finally, known groups validity was also supported. At Week 3 patients receiving ferumoxytol and those with higher Hgb levels reported lower levels of fatigue than those receiving placebo or with lower Hgb levels. Again, known groups validity would have been best conducted based on a more concrete assessment of known fatigue, such as a patient global impression of concept (i.e. patients who categorized their fatigue as mild, moderate or severe). However, given the statistically significant group differences reported, based on a biomarker and treatment arm after only 3 treatment doses, these results could be considered a conservative assessment of the FACIT-fatigue scales ability to distinguish between groups.

As suggested above, a potential limitation of this study was the *post hoc* nature of the psychometric analysis based on clinical trial data. This meant the analyses were confined to the instruments collected as part of the clinical trials and were not included a priori for the purposes of conducting a validation study. Thus, while the available measures were considered appropriate, the responsiveness and known groups analysis for example may have been improved with the inclusion of a patient global impression of concept item. However, clinical trial data is frequently used to provide evidence, or lack thereof, of the measurement properties of new or existing instruments in a particular population [38-40]. Replication of these findings in a prospective validation specific study could still add to the body of evidence supporting the use of the FACIT-fatigue in an IDA population.

The present study suggests the FACIT-fatigue is a conceptually relevant scale to be used in IDA populations, and that its content is clear and meaningful to patients. Further, the psychometric evidence supports the measurement properties of the FACIT-fatigue in IDA populations demonstrating stability of scores over time, internal consistency of items, evidence that it does measure the concept it purports to measure, and sensitivity to detect change. These combined findings support the use of the FACIT-fatigue in IDA populations and further highlight the value of capturing patient-reported outcomes as well as biomarkers in research and clinical settings.

## References

[1] Killip S, Bennett JM, Chambers MD (2007). Iron deficiency anemia. Am Fam Physician.

[2] Looker AC, Dallman PR, Carroll MD, Gunter EW, Johnson CL (1997). Prevalence of iron deficiency in the United States. JAMA.

[3] Cavill I, Auerbach M, Bailie GR, Barrett-Lee P, Beguin Y, Kaltwasser P (2006). Iron and the anaemia of chronic disease: a review and strategic recommendations. Curr Med Res Opin.

[4] World Health Organization (1992). Prevalence of Anemia in Women.

[5] Clark SF (2008). Iron deficiency anemia. Nutr Clin Pract.

[6] Rosenzweig PH, Volpe SL (1999). Iron, thermoregulation and metabolic rate. Crit Rev Food Sci Nutr.

[7] Miller JF (2013). Iron deficiency anemia: a common and curable disease. Cold Spring Harb Perspect Med.

[8] Cella D, Kallich J, McDermott A, Xu X (2004). The longitudinal relationship of hemoglobin, fatigue and quality of life in anemic cancer patients: results from five randomized clinical trials. Ann Oncol.

[9] Lind M, Vernon C, Cruickshank D, Wilkinson P, Littlewood T, Stuart N (2002). The level of haemoglobin in anaemic cancer patients correlates positively with quality of life. Br J Cancer.

[10] Jacobsen PB, Garland LL, Booth-Jones M, Donovan KA, Thors CL, Winters E (2004). Relationship of hemoglobin levels to fatigue and cognitive functioning among cancer patients receiving chemotherapy. J Pain Symptom Manage.

[11] Cella D, Lai JS, Chang CH, Peterman A, Slavin M (2002). Fatigue in cancer patients compared with fatigue in the general United States population. Cancer.

[12] Yellen SB, Cella D, Webster K, Blendowski C, Kaplan E (1997). Measuring fatigue and other anemia-related symptoms with the Functional Assessment of Cancer Therapy (FACT) measurement system. J Pain Symptom Manage.

[13] Kosinski M, Gajria K, Fernandes AW, Cella D (2013). Qualitative validation of the FACIT-Fatigue scale in systemic lupus erythematosus. Lupus.

[14] Lai JS, Beaumont JL, Ogale S, Brunetta P, Cella D (2011). Validation of the functional assessment of chronic illness therapy-fatigue scale in patients with moderately to severely active systemic lupus erythematosus, participating in a clinical trial. J Rheumatol.

[15] Cella D, Yount S, Sorensen M, Chartash E, Sengupta N, Grober J (2005). Validation of the Functional Assessment of Chronic Illness Therapy Scale relative to other instrumentation in patients with rheumatoid arthritis. J Rheumatol.

[16] Chandran V, Bhella S, Schentag C, Gladman DD (2007). Functional assessment of chronic illness therapy-fatigue scale is valid in patients with psoriatic arthritis. Ann Rheum Dis.

[17] Signorovitch J, Brainsky A, Grotzinger KM (2011). Validation of the FACIT-Fatigue subscale, selected items from FACT-thrombocytopenia, and the SF36v2 in patients with chronic immune thrombocytopenia. Qual Life Res.

[18] Hagell P, Hoglund A, Reimer J, Eriksson B, Knutsson I, Widner H (2006). Measuring fatigue in Parkinson’s disease: a psychometric study of two brief generic fatigue questionnaires. J Pain Symptom Manage.

[19] U.S. Department of Health and Human Services Food and Drug Administration (2009). Guidance for industry: patient-reported outcome measures: use in medical product development to support labeling claims.

[20] Rothman M, Burke L, Erickson P, Leidy NK, Patrick DL, Petrie CD (2009). Use of Existing Patient-reported Outcome (PRO) Instruments and their modification: The ISPOR good research practices for evaluating and documenting content validity for the use of existing instruments and their modification PRO task force report. Value Health.

[21] Vadhan-Raj S, Strauss W, Ford D, Bernard K, Boccia R, Li J (2014). Efficacy and safety of IV ferumoxytol for adults with iron deficiency anemia previously unresponsive to or unable to tolerate oral iron. Am J Hematol.

[22] Ware JE, Sherbourne CD (1992). The MOS 36-item short-form health survey (SF-36): I. conceptual framework and item selection. Med Care.

[23] Patrick DL, Gagnon DD, Zagari MJ, Mathijs R, Sweetenham J, Epoetin Alfa Study Group (2003). Assessing the clinical significance of health-related quality of life (HrQOL) improvements in anaemic cancer patients receiving epoetin alfa. Eur J Cancer.

[24] McHorney CA, Ware JE, Raczek AE (1993). The MOS 36-item Short-Form Health Survey (SF-36): II. Psychometric and clinical tests of validity in measuring physical and mental health constructs. Med Care.

[25] Maxwell C (1978). Sensitivity and accuracy of the visual analogue scale: a psycho-physical classroom experiment. Br J Clin Pharmacol.

[26] Joffe H, Yardley L, Marks D, Yardley L (2004). Content and thematic analysis. Research Methods for Clinical and Health Psychology.

[27] Braun V, Clarke V (2006). Using thematic analysis in psychology. Qual Res Psychol.

[28] Willis GB (2005). Cognitive interviewing: a tool for improving questionnaire design.

[29] Morse JM (1995). The significance of saturation. Qual Health Res.

[30] Kerr C, Nixon A, Wild D (2010). Assessing and demonstrating data saturation in qualitative inquiry supporting patient-reported outcomes research. Expert Rev Pharmacoecon Outcomes Res.

[31] McHorney CA, Tarlov AR (1995). Individual patient monitoring in clinical practice: are available health status surveys adequate?. Qual Life Res.

[32] Cronbach LJ (1951). Coefficient alpha and the internal structure of tests. Psychometrika.

[33] Nunnally J (1978). Psychometric Theory.

[34] Scientific Advisory Committee of the Medical Outcomes Trust (2002). Assessing health status and quality-of-life instruments: attributes and review criteria. Qual Life Res.

[35] Kline P (1986). A handbook of test construction: introduction to psychometric design.

[36] Nunnally JC, Bernstein IH (1994). Psychometric theory.

[37] Wyrwich K, Norquist J, Lenderking W, Acaster S (2013). Methods for interpreting change over time in patient-reported outcome measures. Qual Life Res.

[38] Acaster S, Swinburn P, Wang C, Stemper B, Beckmann K, Knappertz V. et.al.: Can the Functional Assessment of Multiple Sclerosis adapt to changing needs? A psychometric validation in patients with clinically isolated syndrome and early relapsing–remitting multiple sclerosis. *Multiple Sclerosis Journal*; 2011;17:1504-1513. 10.1177/1352458511414039 21757536

[39] Lloyd AJ, Loftus J, Turner M, Lai G, Pleil A (2013). Psychometric validation of the visual function questionnaire-25 in patients with diabetic macular edema. Health Qual Life Outcomes.

[40] Prins MH, Guillemin I, Gilet H, Gabriel S, Essers B, Raskob G (2009). Scoring and psychometric validation of the Perception of Anticoagulant Treatment Questionnaire (PACT-Q). Health Qual Life Outcomes.

